# Diagnostic pitfalls of hyperprolactinemia: the importance of sequential pituitary imaging

**DOI:** 10.1186/1756-0500-7-555

**Published:** 2014-08-20

**Authors:** Tomohiro Kawaguchi, Yoshikazu Ogawa, Teiji Tominaga

**Affiliations:** Department of Neurosurgery, Kohnan Hospital, 4-20-1 Nagamachi Minami, Taihaku-ku, Sendai, Miyagi, 982-8523 Japan; Department of Neurosurgery, Tohoku University Graduate School of Medicine, Sendai, Miyagi, Japan

**Keywords:** Dopamine agonist, Hyperprolactinemia, Infertility, Magnetic resonance imaging, Non-functioning pituitary adenoma

## Abstract

**Background:**

The purpose of this study is to confirm whether the serum prolactin cut-off value is definitive to distinguish prolactinoma and non-functioning pituitary adenoma with hyperprolactinemia. We retrospectively reviewed patients with non-functioning pituitary adenoma, including gonadotroph cell adenoma, null cell adenoma and prolactinoma who were surgically treated at Kohnan hospital between June 2005 and March 2012. The patients without endocrinological/neurological symptom and with the tumor larger than 40 mm in diameter were excluded. According to previously reported cut-off value of serum prolactin, mild hyperprolactinemia, which is considered non-definitive (border zone) concentration between prolactinoma and non-functioning pituitary adenoma, were defined as 90 – 200 ng/ml. Ninety-five prolactinoma patients and 212 patients with non-functioning pituitary adenoma were analyzed. The serum prolactin concentration, tumor size, and clinical characteristics were statistically compared.

**Results:**

Receiver operating characteristic (ROC) curve analysis was performed, indicating that cut-off value of serum prolactin concentration to distinguish between non-functioning pituitary adenoma and prolactinoma was 38.6 ng/ml. Although it was statistically good accuracy (the area under the curve; 0.96, sensitivity; 0.99 and specificity; 0.81), the result did not fit the clinical situation as many false-positive cases (40 of 212, 18.9%) were included. Among them, mild hyperprolactinemia were shown in 9 (4.2%) and 53 (55.8%) non-functioning pituitary adenoma and prolactinoma, respectively. Four of 9 border zone patients with non-functioning pituitary adenoma were initially treated with dopamine agonists. Sequential head magnetic resonance imaging revealed no tumor shrinkage in all of them despite serum prolactin concentration was decreased. Surgery was chosen for them 24.6 months in average after the introduction of medication.

**Conclusions:**

Non-negligible number of patients with non-functioning pituitary adenoma presented unexpectedly high concentration of prolactin, fraught with a potential risk of misdiagnosis. While this equivocal population is not the majority, the prolactin cut-off value is not safely applicable. Especially for the patients with border zone prolactin concentration, meticulous follow up with sequential pituitary imaging is important.

## Background

Hyperprolactinemia has been associated with a large number of etiologies, such as certain medication, autoimmune disease, and sellar tumors. Pituitary adenoma is one of the most frequent causes of hyperprolactinemia, and prolactinoma accounts for a high proportion of hyperprolactinemia due to prolactin overproduction and oversecretion. Non-functioning pituitary adenoma is another etiology of hyperprolactinemia, which is induced by compression of the pituitary stalk.

Medical treatment with dopamine agonists (DAs) is highly effective for most cases, so that is widely accepted as the first line of treatment for hyperprolactinemia. In spite of the strong suppression of serum prolactin level, DAs usage has several disadvantages when used for patients with prolactinoma. Intolerance and residence have been reported in some patients [1, 2], and DAs are reported as a potential predisposing factor for pituitary apoplexy [3]. For the pregnant patients, less data is available about the effects of continuous DAs usage on fetal development [4, 5]. Furthermore, DAs can normalize the serum prolactin level in patients with non-functioning pituitary adenoma presented with hyperprolactinemia by inhibition of the normal pituitary function, but there is no chance for tumor regression. So surgical treatment can be a considerable treatment option for some patients with large pituitary adenoma presented with hyperprolactinemia.

To decide the treatment options, discrimination of the cause for hyperprolactinemia is important. Small tumors presented with hyperprolactinemia could be considered prolactinoma in majority of the cases, so that it is not so difficult making correct diagnosis. However, differential diagnosis of large non-functioning pituitary adenoma and prolactinoma is sometimes very difficult despite several endocrinological loading tests and radiographical assessments have been evaluated [6]. Although recent reports indicated the endocrinological discrimination of non-functioning pituitary adenoma from prolactinoma, the cut-off value of serum prolactin concentration between them varied widely from 94 to 200 ng/ml and there are no definitive diagnostic criteria [6–8].

In this study, we retrospectively reviewed the patients with prolactinoma and non-functioning pituitary adenoma presented with hyperprolactinemia to confirm whether the serum prolactin cut-off value is definitive to distinguish them. The characteristics of clinical course and management for these patients were discussed.

## Methods

### Participants

We retrospectively reviewed patients with non-functioning pituitary adenoma, including gonadotroph cell adenoma and null cell adenoma, and prolactinoma who were surgically treated at Kohnan hospital between June 2005 and March 2012. Most patients with prolactinomas were referred from the endocrinological or gynecological clinics. During this period, 724 cases were treated with transsphenoidal surgery, performed by single surgeon (Y.O.). Among them, 95 (13.1%) patients with prolactinoma and 212 (29.3%) patients with non-functioning pituitary adenoma were analyzed.

### Surgical indication for prolactinoma

Surgery was indicated for the patients who required skull base reconstruction to prevent cerebrospinal fluid leakage, and who already presented severe visual disturbance. Patients with small tumor without endocrinological and neurological symptoms were not included. Otherwise, we propose both medical treatment and surgery as treatment options. The advantage and disadvantage of them were equivalently explained. Surgery was performed for patients who understood and chose surgical treatment by themselves. Informed consent was obtained from each patient or guardian on admission, and prior to surgery.

### Data collection

The following variables were recorded in a database and analyzed: age, sex, histological diagnosis, pretreatment serum concentration of anterior pituitary hormones including prolactin, and the maximum diameter of the tumors. Normal range of serum prolactin concentration in our hospital was 4.29 - 13.69 ng/ml in men, 4.91 - 29.32 ng/ml in premenopausal women, and 3.12 - 15.39 ng/ml in postmenopausal women.

### Clinical management

On admission, the general physiological examination and magnetic resonance (MR) imaging were performed and basal value of serum anterior pituitary hormones were examined prior to surgery in all patients. If patients had already treated with dopamine agonist, administration was stopped several weeks before the surgery. In such cases, preoperative highest value of serum prolactin was considered as the initial prolactin value. After required examination, transsphenoidal adenomectomy was performed and the surgical specimens were proceeded for histopathological examination. Pathology review was performed based on World Health Organization classification 2004 [9].

### Determination of the cut-off value of serum prolactin concentration and definition of border zone patients with mild hyperprolactinemia

To identify the cut-off value of serum prolactin level, receiver operating characteristic (ROC) curve analysis was performed with JMP-pro software (SAS Institute Japan, Tokyo, Japan). According to the previous reports, mild hyperprolactinemia, which is considered non-definitive concentration between prolactinoma and non-functioning pituitary adenoma, were defined as 90 – 200 ng/ml [8, 10, 11]. The patients with serum prolactin concentration in this range were considered as border zone cases.

### Statistical analysis

The means and standard deviations (SDs) for serum prolactin concentration was calculated and compared with Student’s *t*-test or Fisher’s exact test. Probability values < 0.05 were considered statistically significant. SPSS software (IBM Japan, Tokyo, Japan) were used for statistical analyses.

## Results

### Patient characteristics

The characteristics of patients with non-functioning pituitary adenoma and prolactinoma were summarized in Table 1. The age of patients with non-functioning pituitary adenoma was higher than with prolactinoma (p < 0.001). The tumor size of non-functioning pituitary adenoma was larger than prolactinoma (p < 0.001). Preoperative serum prolactin concentrations was 28.4 ± 32.1 and 747.0 ± 1548.9 ng/ml (mean ± SD) in patients with non-functioning pituitary adenoma and prolactinoma, respectively. There was statistically significant difference in the mean value of prolactin between the two groups (p < 0.001).

**Table 1:** **Demographic characteristics of the total study population**

Characteristic		Non-functioning adenoma	Prolactinoma	P value
Age, years old	Range (median)	23-80 (60)	14-82 (38)	< 0.001*
Sex	Male (%)	98 (46.2)	20 (21.1)	0.05
	Female (%)	114 (53.8)	75 (78.9)	-
Tumor size	Maximum Diameter (mean ± SD, mm)	23.4 ± 6.3	15.4 ± 9.0	< 0.001*
Serum prolactin concentration	(mean ± SD, ng/ml)	28.4 ± 32.1	747.0 ± 1548.9	< 0.001*

*statistically significant.

SD, standard deviation.

### Serum prolactin concentration and patient distribution

**Figure 1:**
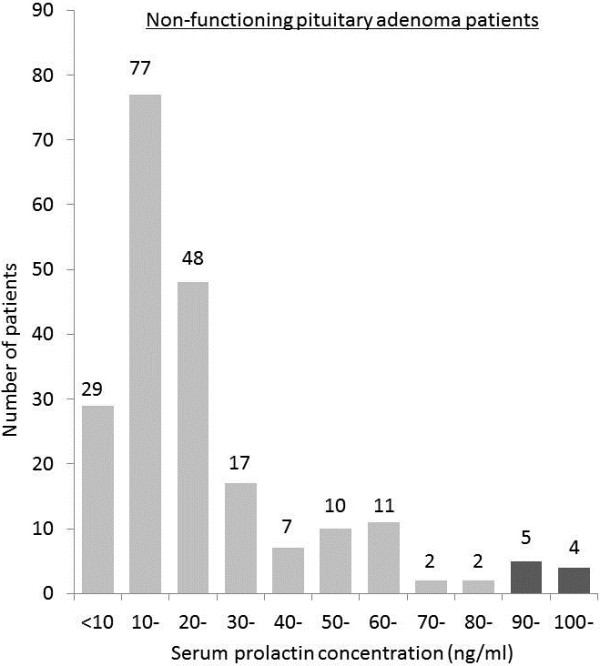
**Frequency distribution according to serum prolactin concentration in patients with non-functioning pituitary adenoma.** Serum prolactin concentration in most patients was around 10 to 20 ng/ml. Nine (4.2%) patients presented the serum prolactin concentration higher than 90 ng/ml (the dark bar graph).

**Figure 2:**
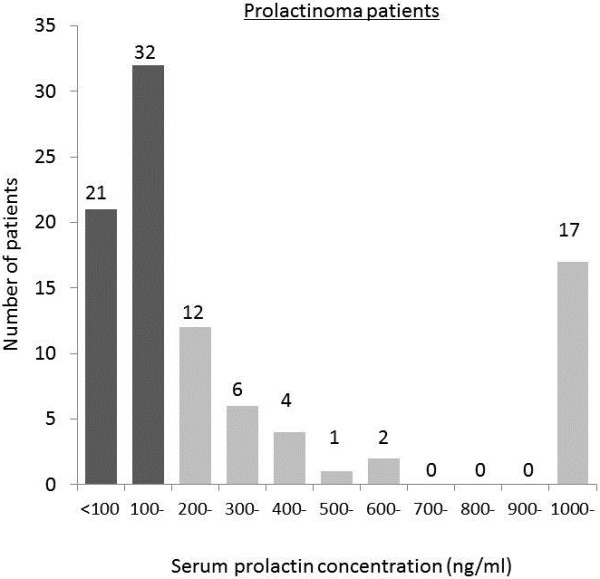
**Frequency distribution according to serum prolactin concentration in patients with prolactinoma.** Serum prolactin concentration in most patients was around 100 ng/ml. Fifty-three (55.8%) patients presented the serum prolactin concentration lower than 200 ng/ml (38.56 – 197.7 ng/ml) (the dark bar graph).

The patient distribution according to serum prolactin concentration was shown in Figures 1 and 2. In 212 patients with non-functioning pituitary adenoma, most patients presented between 10 to 20 ng/ml of serum prolactin concentration and the highest prolactin value was 284.9 ng/ml (Figure 1). In 95 patients with prolactinoma, most of them presented serum prolactin concentration around 100 ng/ml and 53 (55.8%) of them presented the serum prolactin concentration less than 200 ng/ml. Seventeen (18.0%) patients presented the serum prolactin concentration over 1000 ng/ml resulting in raising the mean value (Figure 2).

### Determination of the cut-off value of serum prolactin concentration

**Figure 3:**
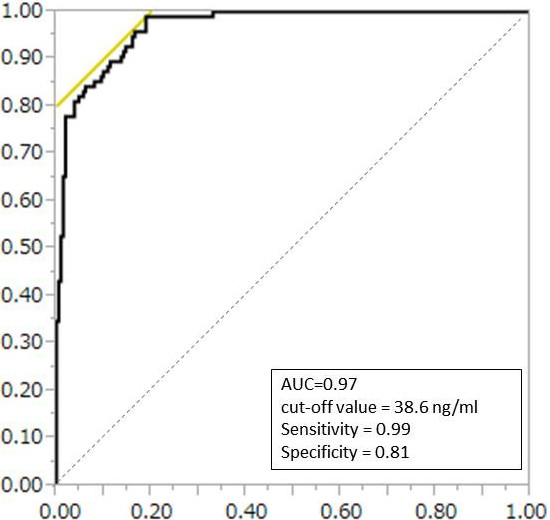
**Receiver operating characteristic (ROC) curve analysis shows that serum prolactin concentration has a high accuracy for differentiating between prolactinoma and non-functioning pituitary adenoma with hyperprolactinemia.**

ROC curve analysis was performed for all cases (Figure 3). The area under the curve was 0.96, which indicated high accuracy of diagnostic value. According to this analysis, cut-off value of serum prolactin concentration to distinguish between non-functioning pituitary adenoma and prolactinoma was 38.6 ng/ml. Sensitivity and specificity were 0.99 and 0.81, respectively. However, this result did not fit the clinical situation, because 40 (18.9%) of 212 patients with non-functioning pituitary adenoma were false-positive. We further analyzed patients whose serum prolactin concentration was between 90 and 200 ng/ml, according to previous reports [8, 10, 11]. This range was considered as the border zone of differential diagnosis.

### Characteristics of patients with mild hyperprolactinemia (Table 2)

**Table 2:** **Characteristics of patients with mild hyperprolactinemia**

Characteristic		Non-functioning adenoma (PRL >90 ng/ml)	Prolactinoma (PRL <200 ng/ml)	P value
Number of patients		9	53	
Age, years old	Range (median)	35-67 (39)	14-82 (34)	0.12
Tumor size	Maximum Diameter (mean ± SD, mm)	22.0 ± 6.0	12.2 ± 6.8	0.01*
Serum prolactin concentration	(mean ± SD, ng/ml)	141.3 ± 68.2	144.5 ± 31.9	0.84

PRL = prolactin.

*statistically significant.

SD, standard deviation.

Nine (4.2%) patients with non-functioning pituitary adenoma and 53 (55.8%) prolactinoma patients were considered as the border zone cases for differential diagnosis. Table 2 showed the summary of these them. There were no statistical differences in serum prolactin concentration (141.3 ± 68.2 vs. 144.5 ± 31.9, p = 0.84), and age (39 vs. 34, p = 0.12). Tumor size was smaller in prolactinoma group (22.0 ± 6.0 vs. 12.2 ± 6.8, p = 0.01).

### Non-functioning pituitary adenoma treated with dopamine agonist

Among the patients with non-functioning pituitary adenoma, 4 patients were initially treated with dopamine agonist. The maximum tumor diameter ranged 10 to 31 mm. Serum prolactin concentration before medication ranged 62.41 to 130 ng/ml and was well suppressed in other 3 patients except for one patient who did not continue medication for some reason. All of them were followed with MR imaging every 6 months. Nevertheless, tumor shrinkage was not achieved in three patients or visual disturbance was aggravated in one patient in her clinical course.

## Discussion

DAs are the first line of treatment for hyperprolactinemia, which is one of the most frequent endocrinological disorders, especially in young women. As the patients present with gonadal dysfunctions and resulted in infertility [6, 12], treatment for hyperprolactinemia is quite important in clinical aspect [13]. DAs have strong suppressive effects on serum prolactin level regardless of etiology, so they tend to be used for both patients with prolactinoma and non-functioning pituitary adenoma with hyperprolactinemia. The mechanism of DAs is quite specific for prolactin normalization. The agents act by binding to specific dopamine receptors on the prolactin-secreting cells, resulting in reduced synthesis and secretion of prolactin, and adenoma cell size [14]. Thus, treatment with DAs can decrease tumor size in about 90% of patients with prolactinoma [15]. In addition, the prolactin-inhibiting mechanism can affect not only adenoma cells but also normal prolactin-secreting cells. So, DAs can normalize the serum prolactin concentration in patients with non-functioning pituitary adenoma, but there is no chance for tumor regression. In clinic, patients with “high” concentration of serum prolactin are usually started treatment with DAs. In this study, we confirmed whether the serum prolactin cut-off value is reasonable to identify who should be treated with DAs.

First, we performed the ROC curve analysis to assess the cut-off value of serum prolactin concentration. This statistical analysis showed good correlation between serum prolactin level and prolactinoma diagnosis. The cut-off value was 38.6 ng/ml with acceptable sensitivity and specificity. However, this result did not fit the clinical situation, as many false-positive cases were included. Such cases have a risk of inappropriate treatment with DAs. The problem still remains to discriminate prolactinoma from non-functioning pituitary adenoma simply with serum prolactin cut-off value.

Previously, several studies have been examined the threshold of serum prolactin concentration to distinguish them. Karavitaki et al. advocated the cut-off value of serum prolactin was 94 ng/ml [8]. Other reports showed that the serum prolactin values were less than 200 ng/ml in most patients with non-functioning pituitary adenoma [10, 11]. Compared to them, 9 (4.2%) patients in our series presented the serum prolactin concentration higher than 90 ng/ml in non-functioning pituitary adeonmas, and 53 (55.8%) of prolactinoma patients presented mild hyperprolactinemia less than 200 ng/ml. Strict attention has to be paid for this border zone group. In our series, 4 patients with non-functioning pituitary adenoma were initially treated with dopamine agonist. All of them were in the border zone group, whose serum prolactin concentration before medication ranged 62.41 to 130 ng/ml.

Taken together, the serum prolactin cut-off value is not safely applicable to establish a correct diagnosis in patients with large pituitary adenoma, nevertheless tumor size is smaller in prolactinoma than non-functioning pituitary adenoma in general population and border zone patients (Tables 1 and 2). As mentioned above, DAs were introduced to 4 patients with non-functioning pituitary adenoma for initial treatment. They showed good result of serum prolactin reduction but no effects on tumor shrinkage. As the monitoring of serum prolactin alone is not sufficient to evaluate the treatment efficacy in such cases, meticulous radiographical follow up is quite important.

Another principle is when to decide the surgical indication. Olafsdottir et al. reported that more than 90% of prolactinoma decreased in size at least 24 months treatment with dopamine agonist [16]. They also reviewed that longer usage of dopamine agonists showed with higher response rate for serum prolactin normalization and in patients with prolactinoma treated with cabergoline, 75 to 100% of prolactin normalization was achieved within 24 months. In our series, patients with non-functioning pituitary adenoma who were treated with dopamine agonist did not show tumor shrinkage, and surgery underwent for them 24.6 months after introduction of medication, on average. Moreover, the usage of dopamine agonists is one of the risk factors of pituitary apoplexy [17–20] and the hemorrhagic event is more frequent within 18 months since the beginning of dopamine agonist treatment [21]. Taken together, patients with hyperprolactinemia who were treated with dopamine agonist should be meticulously followed with MR imaging at least 24 months. When the neurological deterioration or no tumor shrinkage was evident, surgery should be considered.

The present study has several limitations. The patients with pituitary tumor who were successfully treated with dopamine agonist did not include because of lack of histological verification. Also, we did not assess the possibility of macroprolactinemia. However, patients without clinical symptoms were not indicated for any treatment even if the serum prolactin concentration was high, and such cases were not included in this study. More other factors such as sex and tumor size can be considered as the indicative variables for prolactinoma diagnosis. For statistical multivariate analysis, more case accumulation is required. We excluded giant adenomas, because the treatment is still challenging for this entity and outcome is still poor [22]. The treatment strategy is not well established for giant adenomas. More cases are required for better understanding of diagnostic criteria.

## Conclusions

This study indicated that 4.2% of patients with hyperprolactinemia harboring non-functioning pituitary adenoma had a risk of misdiagnosis. As the serum prolactin level could not be definitive, these patients should be followed up with neuroimaging to ensure that the optimum timing for neurosurgical intervention is not missed, even if the primary purpose of gonadal restoration has been achieved with medical treatment. DAs are strong and reliable treatment for patients with hyperprolactinemia, but surgery should be considered for patients with suboptimal results following medical treatment.

### Ethics

The therapeutic protocol was approved by the internal ethics committee of Kohnan Hospital 2013.

## Abbreviations

FSH: Follicle-stimulating hormone
MR: Magnetic resonance
SD: Standard deviation.
